# Cloning-Free Targeting of Endogenous Loci to Generate Fluorescent Reporters in Medaka

**DOI:** 10.21769/BioProtoc.5360

**Published:** 2025-06-20

**Authors:** Simon Knoblich, Kiyoshi Naruse, Ali Seleit, Alexandre Paix

**Affiliations:** 1 Developmental Biology unit, EMBL Heidelberg, Meyerhofstraße, Heidelberg, Germany; 2 Faculty of Medicine, Heidelberg University, Grabengasse, Heidelberg, Germany; 3 National Institute for Basic Biology, Myodaiji Okazaki, Aichi, Japan; 4 Department of Algal Development and Evolution, Max Planck Institute for Biology Tübingen, Max-Planck-Ring, Tübingen, Germany

**Keywords:** Medaka, Bony fish, CRISPR/Cas9, Homology-directed repair, Guide RNA, Fluorescent proteins

## Abstract

CRISPR-Cas9 has democratized genome engineering due to its simplicity and efficacy. Adapted from a bacterial defense mechanism, CRISPR-Cas9 comprises the Cas9 endonuclease and a site-specific guide RNA. In vivo, the Cas9 ribonucleoprotein (RNP) can target specific genomic loci and generate double-strand breaks. Eukaryotic endogenous DNA repair mechanisms recognize the cut site and attempt to repair the DNA either by non-homologous end joining, which introduces insertions/deletions, resulting in a loss of reading frame in coding genes, or through homology-directed repair that maintains the reading frame. The latter approach allows the insertion of fluorescent reporter sequences in frame with protein-coding genes in order to monitor gene expression and protein dynamics in cells and whole organisms. Here, we provide a protocol for targeting endogenous genes to introduce sequences coding for fluorescent reporters in medaka (*Oryzias latipes*). The method is simple, robust, and efficient, thus facilitating straightforward organismal genome editing.

Key features

• Cloning free CRISPR/Cas9 tagging of endogenous genes with fluorescent reporter sequences.

• Guidelines for designing CRISPR/Cas9 endogenous tagging experiments.

• Straightforward generation of transgenic Medaka knock-in reporter lines.

• Versatility with the use of Cas9 mRNA or protein and various fluorescent reporters.

## Graphical overview


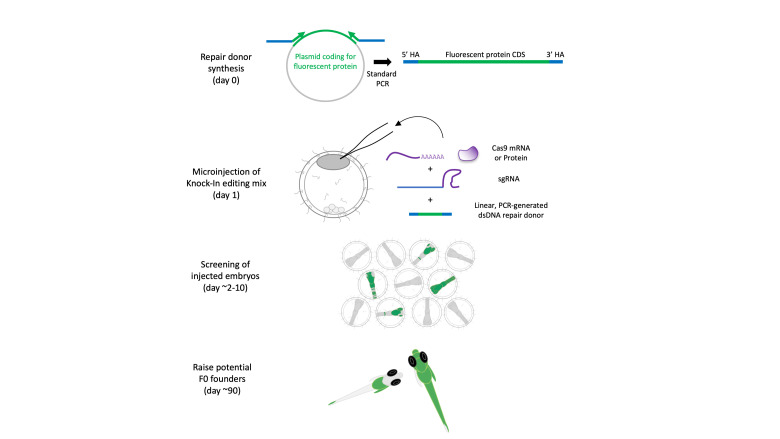


## Background

Bony fish represent a group of vertebrate models that are easy to maintain and manipulate. They include, among others, zebrafish (*Danio rerio*) [1–4], medaka (*Oryzias latipes*) [5,6], cichlid and tilapia fish [7], betta fish [8], and annual killifish [9]. These models have contributed to our understanding of developmental biology and physiological processes and can be used for comparative biology and pharmacological studies. Observing endogenous protein dynamics is critical to understanding morphogenetic processes and signaling pathways during normal and perturbed development. Until recently, routinely generating transgenic lines recapitulating endogenous expression and dynamics has been challenging. The advent of CRISPR/Cas9 genome editing [10–13] and the continuous development of brighter fluorescent reporters [14,15] has changed how genetic and live-imaging approaches can be implemented in bony fish models.

**Figure 1.**
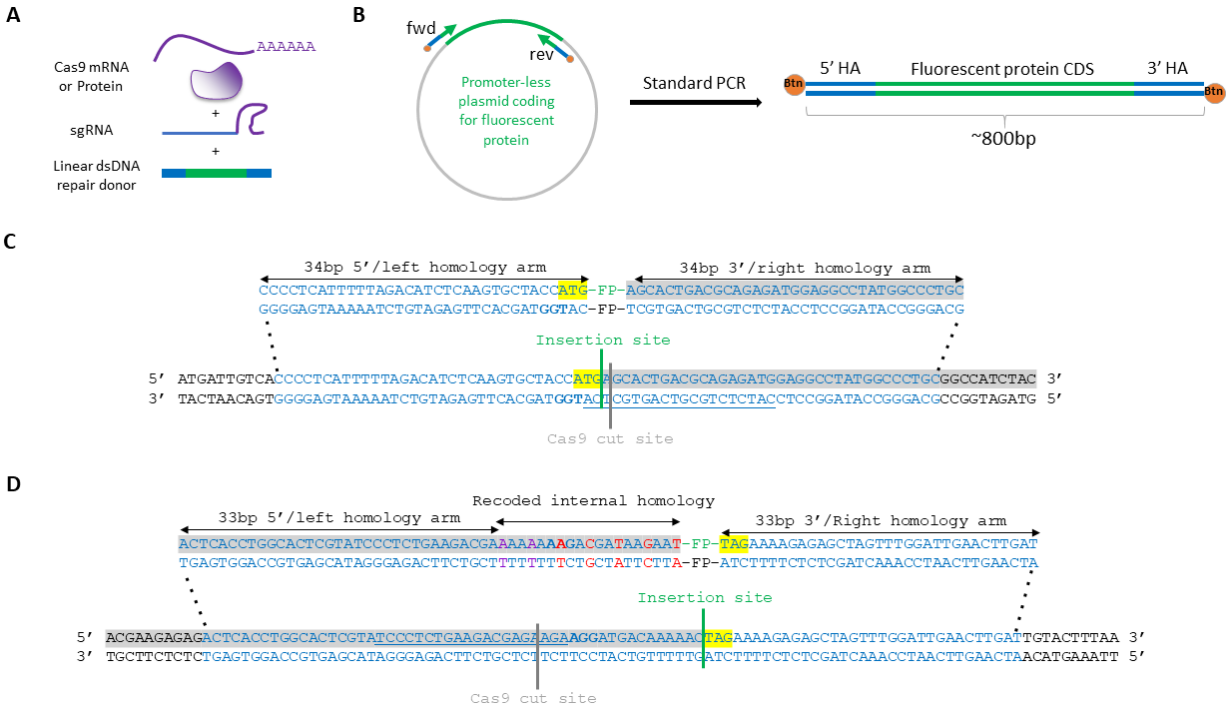
Genome engineering components. (A) Schematic of components for endogenous protein tagging. (B) Schematic of linear dsDNA repair donor PCR amplification with short homology arms (HA) contained in primer sequences. (C) Schematic of homology arms design (blue) for gene tagging with an insertion of a fluorescent protein (FP) reporter downstream of the START codon (yellow, coding sequence in grey), near the Cas9 cut site (PAM marked in bold, guide RNA spacer sequence underlined). (D) Schematic of HA design (blue) for gene tagging with an insertion of a FP reporter upstream of the STOP codon (yellow, coding sequence in grey), and 17 bp downstream of the Cas9 cut site (PAM marked in bold, guide RNA spacer sequence underlined). Synonymous nucleotide changes to prevent Cas9-mediated cutting of the donor/already edited locus are in purple; synonymous changes to reduce internal homology between the donor and the locus are in red.

**Figure 2.**
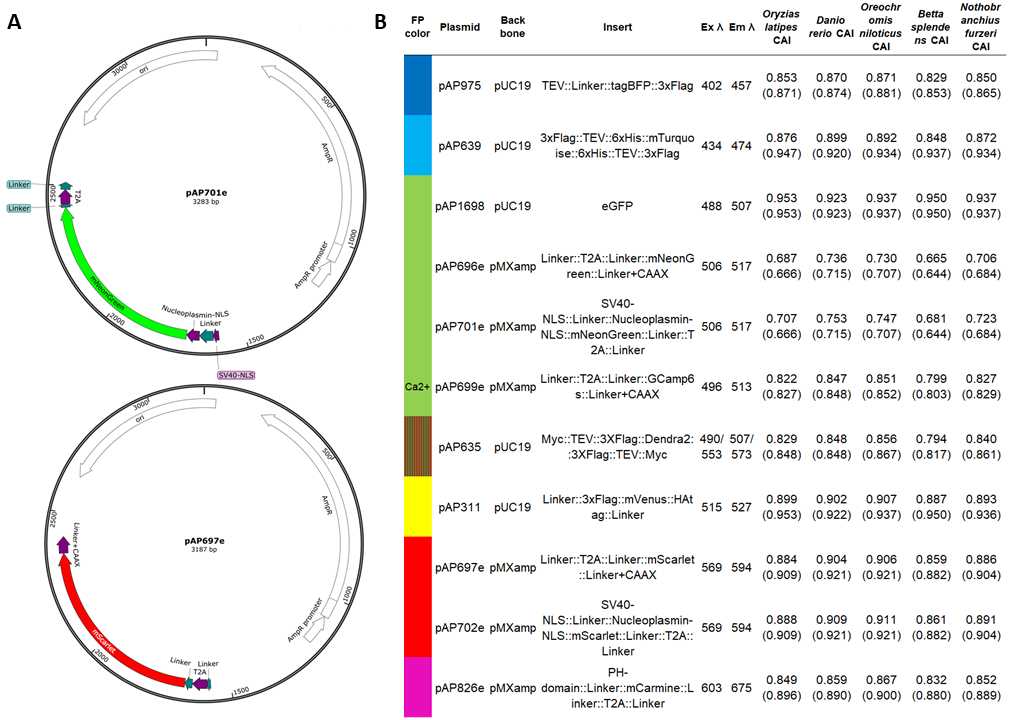
Fluorescent reporter plasmids. (A) Map examples of plasmid templates for PCR repair donor synthesis (done using SnapGene). Plasmids will be submitted to Addgene. (B) Detailed information on plasmid fluorescent protein sequences, tags, and motifs. Codon Adaptation Index (CAI) calculated for the fluorescent protein (FP) sequence, using CAIcal SERVER and codon usage tables from Kazusa DNA Research Institute or HIVE CoCoPUTs [24,25]. We provide the CAI for several established or emergent genetic fish models for future development: *Oryzias latipes* (medaka, Japanese rice fish), *Danio rerio* (zebrafish), *Oreochromis niloticus* (Nile tilapia, cichlid), *Betta splendens* (betta, Siamese fighting fish), and *Nothobranchius furzeri* (turquoise killifish). Maximum excitation and emission wavelengths from FPbase [26]. Dendra2 is a photoconvertible green fluorescent protein (in red), and the excitation and emission wavelengths correspond to the green and red values. Please note that the plasmid can be used to generate various types of donors depending on the PCR primer designs [only the fluorescent reporter, or with ribosomal skipping peptide (T2A) flanked by linkers, membrane localization signal (Linker+CAAX and PH-domain), nuclear localization signal (SV40-NLS and Nucleoplasmin-NLS), peptide and cleavage tags (TEV, 3xFlag, 6xHis and HAtag)].

**Figure 3.**
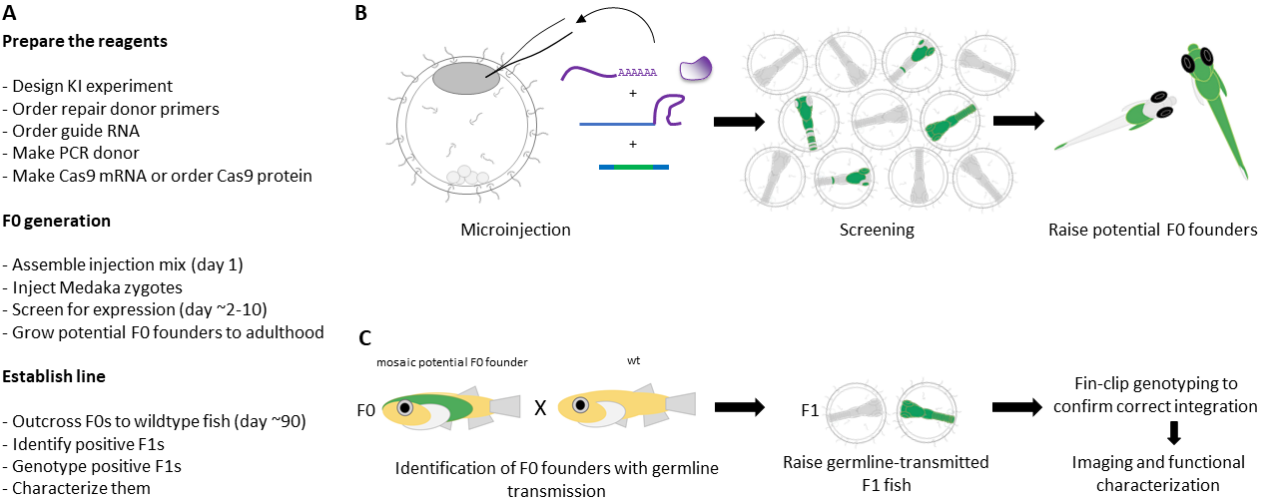
Generation and establishment of endogenously tagged lines. (A) Workflow for generating KI lines. (B) Schematic of F0 generation. (C) Schematic of establishing KI lines.

Here, we present a method to knock in (KI) fluorescent reporters at endogenous gene loci in the Japanese rice fish medaka, an excellent model to study developmental processes and environmental adaptation [16–20]. This method utilizes CRISPR-Cas9 mRNA or protein microinjected together with a linear repair donor coding for a fluorescent reporter with short homology sequences to drive homology-directed repair (HDR). We previously applied this strategy in medaka, *C. elegans*, mammalian cells, and sea anemone [6,21–23]. The method is easy to use and cloning-free, thus reducing the workload associated with custom genetic engineering, and could in theory be applied to other emerging bony fish models (Figures 1, 2, and 3).

## Materials and reagents

**Biological materials**

1. Medaka *(Oryzias latipes)* Cab strain fish

*Note: Other medaka strains can be used (e.g., HdrR). Medaka are maintained as closed stocks in a fish facility built according to welfare standards and directives.*

**Reagents**

1. Sodium chloride (NaCl) (Sigma, catalog number: 1.06404)

2. Potassium chloride (KCl) (Sigma, catalog number: P9541)

3. Calcium chloride dihydrate (CaCl_2_·2H_2_O) (Sigma, catalog number: C3881)

4. Magnesium sulfate heptahydrate (MgSO_4_·7H_2_O) (Sigma, catalog number: 63138)

5. HEPES pH 7.5, 1 M, adjusted to pH 7.5 with NaOH (biomol GmbH, catalog number: 05288)

6. TRIS (Trizma base) (Sigma, catalog number: T1503)

7. Ethylenediaminetetraacetic acid (EDTA) (Sigma, catalog number: E9884)

8. 20% sodium dodecyl sulfate solution (SDS), 20% in H_2_O (Sigma, catalog number: 05030)

9. Proteinase K (VWR, catalog number: 1.24568.0100), resuspended in nuclease-free H_2_O to a concentration of 20 mg/mL, stored as 100 μL aliquots at -20 °C

10. Tricaine (Sigma, catalog number: E10521)

11. Cas9 plasmid for generation of Cas9 mRNA (pCS2-Cas9, can be ordered from Addgene, https://www.addgene.org/47322/)

12. NotI-HF restriction enzyme (NEB, catalog number: R3189)

13. QIAquick PCR Purification kit (Qiagen, catalog number: 28104)

14. Nuclease-free H_2_O, non-DEPC-treated (Thermo Fisher, catalog number: AM9938)

15. mMESSAGE mMACHINE SP6 In Vitro Transcription kit (Thermo Fisher, catalog number: AM1340)

16. RNeasy Mini kit (Qiagen, catalog number: 74104)

17. Glycerol-free recombinant SpyCas9 protein (IDT, catalog number: 10007806); for storage conditions, see step A2

18. Synthetic sgRNA [Sigma, Custom CRISPR, Synthetic RNA, spCas9, sgRNA (crRNA + tracrRNA as one), 3 nmol, not modified, standard purification, dry]

19. 5′-Biotinylated primers for amplification of linear dsDNA donor (Sigma, Custom Oligo, DNA-Oligos in tubes, 5′ Modification Biotin, 0.025 μmol, desalted, at 100 μM in H_2_O)

20. Fluorescent reporter plasmids (Figure 2)

21. Q5 2× PCR master mix (NEB, catalog number: M0492S) or Phusion 2× PCR master mix with HF buffer (NEB, catalog number: M0531S)

22. MinElute PCR Purification kit (Qiagen, catalog number: 28004)

23. MinElute Gel Extraction kit (Qiagen, catalog number: 28604)

24. 6× Orange G loading dye (NEB, catalog number: B7022S)

25. Sigmacote siliconizing reagent (Sigma, catalog number: SL2-25ML), stored at 4 °C in the dark

26. Agarose (Sigma, catalog number: A9539)

27. Genotyping primers (Sigma, Custom Oligo, DNA-Oligos in tubes, no modification, 0.025 μmol, desalted, at 100 μM in H_2_O)

28. 100 bp DNA ladder (NEB, catalog number: N3231) and GeneRuler DNA ladder mix (ThermoFisher, catalog number: SM0333)

29. Phenol red solution (Sigma, catalog number: P0290)

**Solutions**

1. Embryo rearing medium (ERM) 100× (see Recipes)

2. Embryo rearing medium (ERM) 1× (see Recipes)

3. Fin-clip buffer (see Recipes)

4. 0.4% (w/v) Tricaine solution (see Recipes)

**Recipes**

**1. Embryo rearing medium (ERM) 100×**

**Table:** 

Component	Final concentration	Quantity or volume
NaCl	1711.1 mM (100.00 g/L)	100 g
KCl	10.2 mM (3.00 g/L)	3 g
CaCl_2_·2H_2_O	27.2 mM (3.02 g/L)	4 g
MgSO_4_·7H_2_O	64.9 mM (7.81 g/L)	16 g

Fill up to 1 L with dH_2_O. Autoclave and store at room temperature (RT).

**2. Embryo rearing medium (ERM) 1×**

**Table:** 

Component	Final concentration	Quantity or volume
100× ERM	17.111 mM (1.0 g/L) NaCl, 0.102 mM (30.0 mg/L) KCl, 0.272 mM (30.2 mg/L) CaCl_2_, 0.649 mM (78.1 mg/L) MgSO_4_	10 mL
1 M HEPES, pH 7.5 (NaOH-adjusted)	16.785 mM (4.0 g/L)	17 mL

Fill up to 1 L with dH_2_O. Autoclave and store at RT.

**3. Fin-clip buffer**

**Table:** 

Component	Final concentration	Quantity or volume
2 M TRIS-HCl, pH 8.0	400 mM TRIS-HCl (48.45 g/L)	100 mL
0.5 M EDTA (NaOH adjusted), pH 8.0	5 mM EDTA (1.46 g/L)	5 mL
5 M NaCl	150 mM NaCl (8.76 g/L)	15 mL
20% (w/v) SDS	0.1% (w/v) SDS (1.00 g/L)	2.5 mL

Use PCR-grade reagents only.

Fill up to 500 mL with dH_2_O, then filter-sterilize and store at RT until use. Should SDS precipitate over time, briefly heat to 60 °C until fully dissolved prior to adding Proteinase K.

Add 50 μL of Proteinase K (20 mg/mL, in dH_2_O) fresh to 1 mL of buffer prior to use.

**4. 0.4% (w/v) Tricaine solution**

**Table:** 

Component	Final concentration	Quantity or volume
Tricaine powder	15.3 mM Tricaine (3.99 g/L)	400 mg
1 M TRIS-HCl at pH 9.0	21 mM TRIS-HCl (2.543 g/L)	2.1 mL

Fill up with dH_2_O to 100 mL, adjust pH to 7.0, then autoclave and store at -20 °C in the dark (light sensitive).

**Laboratory supplies**

1. Eight-PCR-tube strips with thin walls (VWR, catalog number: 93001-118)

2. Gel loading tips, 20 μL (Eppendorf, catalog number: 0030 001.222)

3. Borosilicate capillaries, OD 1 mm, ID 0.58 mm (Harvard Apparatus, catalog number: 30-0016)

## Equipment

1. Tabletop centrifuge (Eppendorf, catalog number: 5425)

2. PCR thermocycler with temperature-gradient functionality (Bio-Rad, model: C1000 Touch)

3. Agarose minigel electrophoresis system (Fisher Scientific, catalog number: E2102-F-E)

4. NanoDrop spectrophotometer (Thermo Fisher Scientific, catalog number: ND-1000)

5. Electronic microinjector (Calibre Scientific, model: FemtoJet 4x Microinjector 5252000025)

6. Capillary needle micromanipulator (Narishige, catalog number: MN-153)

7. Fluorescence screening stereomicroscope (Nikon, catalog number: SMZ18) with fluorescence light source (Lumencor, model: Sola SM 5-LCR-VA)

8. Large working distance injection stereomicroscope with inverted illumination (Olympus, catalog number: SZX7) with base stand light column model (Olympus, catalog number: SZX2-ILLTQ) and objective (Olympus, catalog number: SZX-ACH 1X)

9. Fin clip fine scissors (Fine Science Tools, catalog number: 14061-09)

10. Fin clip tweezers (Fine Science Tools, catalog number: 11252-20)

## Procedure

**A. Cas9 mRNA synthesis and protein storage**

*Notes:*

*1. Initially, we used a Cas9 mRNA containing two nuclear localization signals (NLSs) and a monomeric streptavidin (mSA), but we did not observe a consistent difference with a Cas9 version without the mSA [6]. Although the number and nature of the NLS and linkers fused to Cas9 might improve the cutting efficiency [27,28], we now routinely use a Cas9 mRNA version with one NLS when lethality associated with a particular locus is a concern. For other KI, we use Cas9 protein for higher KI efficiency (Table 1).*

*2. We use commercially available Cas9 recombinant protein shipped in glycerol-free buffer. We observed that this storage buffer, compared to glycerol-based buffer, and together with the capillary siliconization described below, simplifies the microinjection process. For other commercial Cas9 formulations or lab-made Cas9 [22,29], this protocol might need to be customized.*

*3. Cas9 mRNA and RNP are complementary. Cas9 RNP allows DNA cutting soon after injection, improving the genome editing efficiency, reducing the level of mosaicism, and increasing the likelihood of homozygous edits. However, microinjection of RNP-based mixes is more difficult due to higher viscosity and more frequent needle clogging and may generate lethal knock-out (KO) alleles more frequently. On the contrary, mRNA-based mixes are easier to inject but can be less efficient and lead to an increased level of mosaicism, since the mRNA needs to be translated into functional Cas9 protein during embryogenesis [30,31]. In some cases, this can reduce lethality due to KO alleles. We therefore provide both possibilities in this protocol for researchers to be able to maximize their chances of recovering a KI allele.*

*4. Several spyCas9 variants have been reported, with relaxed PAM specificities [32,33]. However, in our experience, only the VQR variants (NGA PAM) have exhibited satisfactory in vivo efficiency (in* C. elegans *and mammalian cells, unpublished results). Cas9 variants comparison in vivo and in vitro have also demonstrated that some variants are less efficient [34,35].*

**Table 1.** Reference numbers for two example injection rounds using Cas9 protein. Comparison of microinjected Cas9 RNP to Cas9 mRNA delivery [6]. Cas9 protein also contains NLS, according to the manufacturer. Out of all positive KI embryos from Cas9 RNP injection (n = 2, injections on separate days using freshly prepared mixes), 22.2% (6/27) and 18.8% (3/16) survived after hatching and can be used to test germline transmission and transgenic line establishment. ± represents the standard deviation from the average.

Source	Targeted KI	Cas9 used	Early lethality (dead after 48h/injected embryos)	Mosaic expression in F0s (positive embryos/surviving injected embryos)	Average early lethality	Average mosaic expression in F0s	Average number of positive embryos per 100 injected embryos
This manuscript	*eGFP-cbx1b*	IDT glycerol-free recombinant Cas9 protein	34.3% (46/134) and 40.2% (45/112)	30.7% (27/88) and 23.9% (16/67)	37.3% (±4.2)	27.3% (±4.8)	17.5
Seleit et al. [6]	*eGFP-cbx1b*	Cas9-mSA mRNA (Nt SV40-NLS + Ct Nucleoplasmin-NLS)	41.0% (16/39) and 51.1% (23/45)	21.7% (5/23) and 13.6% (3/22)	46.0% (±7.1)	17.6% (±5.7)	9.5
Seleit et al. [6]	*eGFP-cbx1b*	Cas9 mRNA (Nt SV40-NLS + Ct Nucleoplasmin-NLS)	36.3% (24/66) and 32.3% (11/34)	14.2% (6/42) and 17.3% (4/23)	34.3% (±2.8%)	15.7% (±2.1)	10
Seleit et al. [6]	*eGFP-cbx1b*	Cas9 mRNA (Ct SV40-NLS)	10.8% (5/46) and 18.8% (10/53)	7.3% (3/41) and 11.6% (5/43)	14.8% (±5.6)	9.4% (±3.0)	8.1

1. Cas9 mRNA synthesis

a. Linearize the Cas9 template plasmid (pCS2-Cas9) for in vitro transcription with NotI-HF [36]. Assemble the following reaction (Table 2).

**Table 2.** Linearization of Cas9 mRNA plasmid

Component	Quantity
pCS2-Cas9 plasmid (Addgene, catalog number #47322)	Around 10 μg
Cutsmart 10× buffer	5 μL
NotI-HF	1.5 μL
Nuclease-free dH_2_O	to 50 μL
Incubate at 37 °C for 3 h	

b. Purify the linearized plasmid using a QIAquick PCR Purification kit as per the manufacturer’s instructions, then measure the concentration on a Nanodrop and dilute the eluted DNA in nuclease-free H_2_O to obtain a concentration of approximately 100 ng/μL.

c. Perform the in vitro transcription reaction using a mMESSAGE mMACHINE SP6 kit (Table 3).

**Table 3.** In vitro transcription of Cas9 mRNA

Component	Quantity
Linearized pCS2-Cas9 template (around 100 ng/μL)	6 μL
2× NTP/Cap buffer	10 μL
10× transcription buffer	2 μL
SP6 enzyme	2 μL
Incubate at 37 °C for 2 h	
TurboDNAse	1 μL
Incubate at 37 °C for another 15 min	

d. Purify the Cas9 mRNA: Purify the Cas9 mRNA using a RNeasy Mini kit as per the manufacturer’s instructions, eluting in 35 μL of nuclease-free H_2_O. Measure concentration and aliquot the required volume for a final concentration of 150 ng/μL in a 10 μL injection mix in individual tubes (for instance, if the concentration is 500 ng/μL, aliquot 3 μL per tube). Store aliquots at -80 °C until use.

2. Storage of Cas9 recombinant protein:

a. Cas9 from IDT (Alt-R S.p. Cas9 V3, glycerol-free, catalog number: 10007806) provided at 10 μg/μL (61 μM), make single-use aliquots of 1 μL per tube in PCR strips.

b. Store at -20 °C until use. Avoid any unnecessary freeze/thaw cycles.

**B. Guide RNA design and ordering**

*Notes:*

*1. Cas9 is an RNA-guided endonuclease requiring a guide RNA to target a specific DNA sequence. In the bacterial defense system, the guide RNA consists of two small RNAs [13]: the CRISPR RNA (crRNA) containing a nucleotide sequence complementary to the targeted DNA (corresponding to the spacer part of the CRISPR locus), and a trans-activating crRNA (tracrRNA) that brings together the Cas9 nuclease and the crRNA. The crRNA and tracrRNA can be combined into a single guide RNA (sgRNA) (Figure 1).*

*2. We use Cas9 from* Streptococcus pyogenes *(Spy), which is the most commonly used, in mRNA or protein delivery form. For the guide RNA, we order synthetic guide RNA from commercial providers, since it is convenient and can be delivered in a few days. Also, it has been reported that they are more efficient and less toxic than guide RNAs produced by transcription [37,38].*

*3. We recommend manually designing guide RNAs since online design tools might not take into account various parameters presented here. For instance, some tools score guide RNAs for in vitro transcription and therefore favor guide RNAs starting with a G for efficient transcription, whereas we use chemically synthesized guide RNAs, which do not require a G at the 5′ end. Similarly, predicted KI scores might not consider short homology arms and therefore might overlook the importance of the distance between the Cas9 cut and the insertion site [39].*

*4. In other model organisms, we did not find that denaturing/renaturing guide RNA, or pre-incubating the Cas9 RNP at 37 °C, improved efficiency [21,22].*

1. Identify the genomic position to target.

a. Using the Ensembl! Genome browser (Japanese medaka HdrR assembly) [40], locate your gene of interest (by name or blast search) on the medaka strain that you are using (the HdrR strain assembled genome is very close to the Cab strain that we use).

b. Find the transcript of the gene (if several splicing isoforms exist, choose the one that you would like to tag).

c. Download the genomic sequence of the transcript (including introns and upstream/downstream sequences).

d. Verify that the transcript model seems complete and not truncated (File S1). This can be done, for instance, by looking at the domain prediction using the translated transcript (using SMART, http://smart.embl-heidelberg.de/), and comparing to human homologues (see HGNC website, https://www.genenames.org) [41,42].

e. Determine where you want to tag the transcript product (Nt, Ct, internal), depending on protein characteristics and previous literature in other models, if available. If no possible Nt/Ct insertion can be determined, or if a specific group of transcripts needs to be tagged, internal tagging (between protein domains) can also be suitable, as long as the insertion does not impair protein domains or motifs [43].

f. Find a guide RNA: the sequence giving the locus specificity of a guide RNA consists of 20 nucleotides (nt) corresponding to the locus to target. For spyCas9, these 20 nt (corresponding to the spacer part of the CRISPR locus) need to be immediately upstream of a protospacer-adjacent motif (PAM), which is the trinucleotide NGG, where N can be any nucleotide. Therefore, to find a guide RNA, a PAM motif has to be identified in the locus to be edited. The PAM can be located in both DNA strands (5′ NGG 3′) (Figure 1).

2. Criteria for guide RNA choice and ordering

a. Guide RNAs with a GC content between 50% and 70% are more efficient, as well those not containing a C at the 3′ end of the protospacer sequence [36,44].

b. However, one of the main factors affecting KI efficiency is the distance between the insertion site and the Cas9 cut (located at -3 nucleotides upstream of the PAM). Therefore, we recommend to always choose the guide RNA generating a Cas9 cut as close as possible to the insertion site, even if more efficient guide RNAs can be identified at larger distances from the insertion site [39,45–47].

c. Potential off-targets can be identified using online tools such as CCTop (https://cctop.cos.uni-heidelberg.de/) or CHOPCHOP (https://chopchop.cbu.uib.no/) [48,49]. However, results should be manually examined based on the number and location of mismatches, taking into consideration that one or two mismatches close to the PAM are enough to reduce the efficiency of Cas9 cutting [50].

d. Order the guide RNA. The 20 nt sequence upstream of a PAM (5′ to 3′, the spacer) is what makes the guide RNA specific. Therefore, it is the sequence that has to be specified when ordering a guide RNA from commercial providers. Most commercial providers accept DNA and RNA entries. We use Sigma as a commercial provider (for ordering specifications, see Materials and reagents). Guide RNAs are ordered as sgRNAs. Upon receipt, briefly spin the tube, add 30 μL of nuclease-free H_2_O to the 3 nmol for a stock concentration of 100 μM (3.2 μg/μL). Carefully mix by pipetting and store at -80 °C. For Cas9 mRNA injection, due to the lower concentration of guide RNA in the injection mix, a working dilution of the sgRNA can be done at 10 μM in nuclease-free H_2_O, and stored for several weeks at -80 °C.

**C. Linear DNA repair donor design and synthesis**

*Notes:*

*1. The position of the insertion site relative to the Cas9-induced cut site will determine the repair donor design. We recommend first designing your construct in silico, making sure that the insertion is in frame (Figure 1, File S1).*

*2. Any conventional fluorescent protein sequence can be used since most of them are less than 1 kb in length (Figure 2, File S2). However, we recommend avoiding large insertions (more than 1.5 kb) since the insertion efficiency is inversely proportional to the size of the insert [45,46]. For larger insertions, other methods can be used [51–56].*

*3. To generate the PCR donor, we recommended using a template plasmid not containing any promoter sequence to avoid traces of plasmid in the purified repair donor driving episomal DNA expression and false positives.*

*4. We provide a list of plasmids to generate inserts with various fluorescence colors (Figure 2, File S2). These plasmids do not contain full expression cassettes and contain additional peptide tags and motifs that can be included in the donors. Just adapt the annealing sequences of the primers to include these sequences. Gene expression reporters can be generated by using 2A ribosomal skipping peptides and localization signals (nucleus or plasma membrane) [57]. Please note that a codon usage index superior to 0.5 has given us satisfactory expression, but we usually aim for 0.7–0.8.*

*5. We use repair donors generated with PCR primers containing 5′ biotin modifications. Previous work has shown that such modification prevents in vivo concatemerization [5]. However, in our hands, this generally did not improve the overall KI efficiency [6]. Since it is conceivable that concatemerization might be problematic for the purpose of KI, we continue to use biotinylated donors, but other users might prefer not to do so.*

1. Identification of homology arms, spanning sequence, and annealing region for PCR primers.

a. The repair donor and edited locus should not be targeted by the guide RNA used to edit the gene of interest. If the insertion is at/around the Cas9 cut site (2 bp window), this will disrupt the guide-RNA targeted sequence and prevent subsequent re-cutting by Cas9. However, it is always important to double-check that the spacer sequence relative to the PAM is disrupted, and to take into consideration that NGA and NAG motifs can act as weak PAMs [33,50].

b. To avoid recreating a PAM sequence after insertion, which could result in re-cutting by Cas9, it is important to include sequence modifications to change the spacer sequence or the PAM. Synonymous mutations can be used or an inclusion of extra nucleotides (a codon) in the insert to avoid recreating a PAM. We therefore recommend designing guide RNAs cutting in coding regions for which it is possible to use synonymous codons in the repair donor to prevent Cas9 cutting and recombination between the Cas9 cut and insert, especially when the insertion is away from the Cas9 cut (see below) [39,45,46].

c. Homology arm length should be around 32–40 bp [6,45,58]. Homology arms are defined as the sequences flanking both the Cas9 cut and the insertion site (or the farthest modification from the Cas9 cut). Any sequence in between the Cas9 cut and the insertion site is not part of the homology arms, but part of the insert. Since this spanning sequence is similar to the endogenous locus and can drive premature recombination, it is important to recode it using synonymous codons to reduce the internal homology and prevent Cas9 cutting. Please note that for such a short stretch of codons, the codon usage score does not matter.

d. PCR repair donors will therefore be generated using forward and reverse primers containing, from 5′ to 3′, the 32–40 nt homology arms, the recoded spanning sequence if required, and the 19–21 nt annealing sequence to the insert (fluorescent reporter and other motifs).

e. Order the primers as salt-free purification at 100 μM in nuclease-free H_2_O and store at -20 °C.

2. Donor synthesis and purification.

a. Assemble the PCR mix as follows (Table 4):

**Table 4.** Repair donor synthesis with single PCR amplification

Component	Quantity
Fluorescent reporter plasmid at ~100 ng/μL (from a standard miniprep)	0.1 μL
Forward PCR primer at 100 μM	0.25 μL
Reverse PCR primer at 100 μM	0.25 μL
2× Phusion or Q5 master mix	25 μL
Nuclease-free H_2_O	24.4 μL
A master mix to run 4–8 PCR amplifications can be performed for higher repair donor yield	

b. In thin-walled PCR tubes, vortex the PCR mix reactions and run the amplification as follows (Table 5):

**Table 5.** Repair donor synthesis PCR parameters

Step	Time and temperature
Initial denaturation	2 min at 98 °C
Denaturation step	30 s at 98 °C
Annealing step	30 s at 61.5 °C
Elongation step	45 s at 72 °C
Repeat denaturation/annealing/elongation steps for a total of 30 cycles	
Final elongation	10 min at 72 °C
Hold at 10 °C	

c. In the vast majority of cases, a PCR annealing temperature of 61.5 °C results in a clean PCR, and 45 s of elongation is enough to amplify the FP and homology arms (~900 bp total). If the PCR leads to nonspecific bands or insufficient donor yield, a PCR gradient can be run to determine the optimal annealing temperature. For this, assemble the following (Table 6):

**Table 6.** Repair donor synthesis with a master mix for PCR amplification

Component	Quantity
Plasmid template at ~100 ng/μL (from a standard miniprep)	0.8 μL
Forward PCR primer at 100 μM	2 μL
Reverse PCR primer at 100 μM	2 μL
2× Phusion or Q5 master mix	200 μL
Nuclease-free H_2_O	195.2 μL
Split into 8×50 μL reactions in PCR tubes	
Run the PCR with a gradient of annealing step temperature between 60 and 72 °C	

d. Adding DMSO at 3% v/v final (1.5 μL in 50 μL reaction) can also improve the PCR reaction yield/specificity. In ~1% of all cases, the PCR amplification fails or is not clean, and a nested PCR will be required; in this case, design a first pair of PCR primers annealing at the ends of the insert sequence and containing 20 nt of the flanking sequence as 5′ overhangs, run a first round of PCR, purify it, and then do a second round of PCR with primers annealing at the ends of the first round PCR product (i.e., at the 5′ ends of the respective previous primer overhang sequences), and containing the remainder of the flanking sequences, in order to get to a repair donor containing full homology arms. Since long primers can be eluted together with the PCR donor during purification, if one of the primers used for the PCR repair donor is over 65 nt long, we recommend doing a nested PCR with a second pair of primers annealing at the extremities of the donors (18–20 nt long). This will reduce the risk of having PCR primers eluted with the PCR donor during purification [58].

e. Run an agarose gel to check the PCR product before or after purification. Use a fast-running loading dye such as Orange-G to avoid dye background at the expected PCR product size.

f. Purify the PCR donors using a MinElute PCR Purification kit and elute in 10 μL of nuclease-free H_2_O. Up to 8 × 50 μL of PCR mix can be pooled into a single column to increase concentration. Measure the concentration on a Nanodrop (usually ~100 ng/μL for the purification of a 50 μL PCR amplification) and store at -20 °C.

g. If the PCR primers used are longer than 65 nt, or if ultrapure donors are desired, the PCR reaction can also be gel-purified using a MinElute Gel Extraction kit, followed by a second cleanup using a MinElute PCR Purification kit, to ensure appropriate DNA quality.

**D. Genome editing mix and injection**

*Notes:*

*1. Medaka embryos fully develop at constant temperatures between 18 and 35 °C. We use 27 °C as a standard temperature, but this can be adapted depending on the strain injected or the gene targeted.*

*2. For injection of Cas9 protein mixes, we routinely siliconize injection capillaries prior to needle pulling (see below), in order to mitigate frequent needle clogging. This is not necessary for mixes without Cas9 protein.*

**Figure 4.**
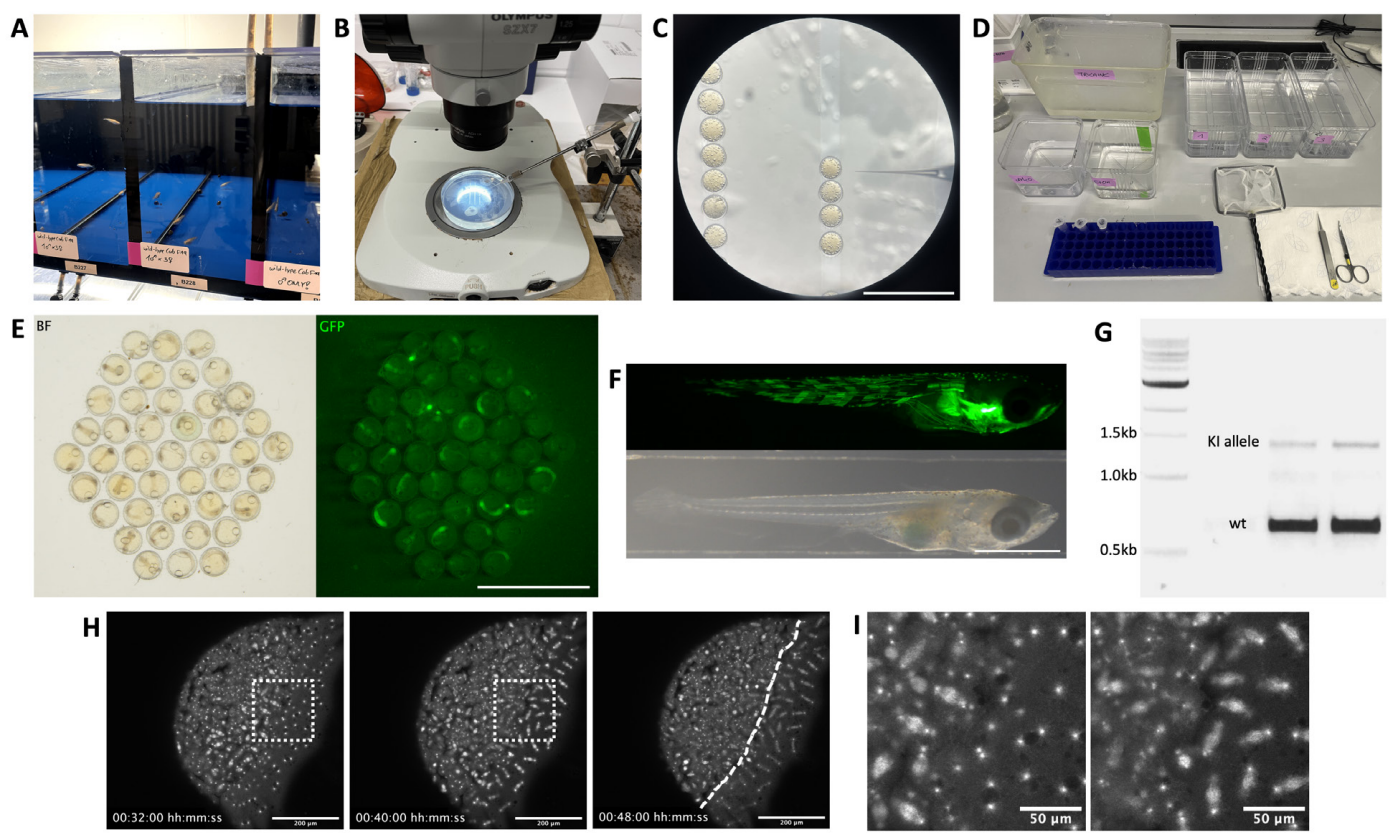
Example images illustrating the steps of KI line generation. (A) Medaka tanks housing wild-type fish crosses of one male and three females for microinjection. Males are placed into a separate tank the day before microinjection and returned prior to injection to allow for synchronous collection of 1-cell stage embryos. (B) Medaka microinjection setup. Embryos are placed and oriented in 1.5% agarose lanes cast using a mold, on top of a large working distance stereoscope with a capillary needle holder connected to a microinjector. (C) Medaka 1-cell stage embryos and microinjection needle viewed under a stereoscope. Scale bar = 5 mm. (D) Materials required for fin-clipping procedure, including, from left to right: a fish tank containing Tricaine anesthetizing agent, containers filled with dH_2_O and EtOH for rinsing of instruments, individual labeled tubes for fin samples, appropriately-sized recovery containers labeled and filled with fish water for housing of individual fish, a net, sharp scissors and forceps. (E) Embryo dish of F0 embryos, one day after microinjection with eGFP-Cbx1b KI injection mix for ubiquitous labeling of nuclei; after removal of dead embryos and prior to screening for possible founders with fluorescence. Left: brightfield (BF) image; right: GFP fluorescence image. Scale bar = 5 mm. (F) An F0 embryo after hatching, previously injected and screened as a potential founder for KI of mNeonGreen into the *myosin-heavy-chain* locus, exhibiting mosaic fluorescence (GFP fluorescence shown above) in muscle tissue regions of the hatchling body (brightfield image shown below). Scale bar = 1 mm. (G) Agarose gel for a genotyping PCR with primers binding outside the insertion locus. The wild-type (wt) allele band for eGFP-Cbx1b is visible as a band of 635 bp, and the KI allele band with insertion of eGFP (714 bp) is visible at 1,349 bp, thus making these two F1s heterozygous for the insertion allele. (H) Example of KI line test imaging, in this case confocal imaging of a *marpe1b(eb1)-mScarlet* KI reporter line [6], labeling microtubule plus-ends. Shown are representative time points of a live imaging timelapse (File S3) in side view of a stage-10 early-blastula medaka embryo, with labeled mitotic spindles visible in both the embryo blastula to the left and the yolk syncytial layer to the right of the dashed line. Scale bar = 200 μm. (I) Magnification of the dashed-line square in H, highlighting the Marpe1b(eb1)-mScarlet labeled mitotic spindles. Scale bar = 50 μm.

*3. For the purpose of establishing stable KI lines, injections should be performed into 1-cell stage embryos as described previously [59] (Figures 3 and 4). In principle, medaka injections can also be carried out at the 2- or 4-cell stage [60], should more mosaic editing be desired.*

1. Genome editing injection mix:

a. Using Cas9 mRNA.

Assemble the following mix (Table 7):

**Table 7.** Genome editing microinjection mix with Cas9 mRNA

Component	Quantity
sgRNA (working dilution at 10 μM or 0.32 μg/μL in nuclease-free H_2_O)	0.5 μL (to a final concentration of 0.5 μM)
Cas9 mRNA	1.5 μg (to a final concentration of 150 ng/μL)
Linear PCR repair donor	100 ng (to a final concentration of 10 ng/μL)
Nuclease-free H_2_O	To a final volume of 10 μL
Centrifuge the mix at 6,000× *g* on a tabletop centrifuge for 5 s and keep on ice for injection within the same day	

b. Using Cas9 protein.

Thaw the following components to RT and assemble the mix in the following order (gently mixing by pipetting in between and avoiding the introduction of bubbles) (Table 8):

**Table 8.** Genome editing microinjection mix with Cas9 recombinant protein

Component	Quantity
Recombinant Cas9 at 10 μg/μL (61 μM)	1 μL (to a final concentration of 1 μg/μL)
sgRNA at 100 μM (3.2 μg/μL)	1.22 μL (to a final concentration of 12.2 μM)
Linear PCR repair donor	400 ng (to a final concentration of 40 ng/μL)
Nuclease-free H_2_O	To a final volume of 10 μL
Centrifuge the mix at 6,000× *g* on a tabletop centrifuge for 5 s and keep at RT for injection within the next 1–2 h.	

c. Injection capillary siliconization.

Load the inside of a borosilicate capillary (prior to needle-pulling) with 20 μL of Sigmacote reagent under a chemical fume hood, using a gel loading pipette tip shortened with scissors for easier handling. Tilt the capillary back and forth to swirl the reagent through the entire inside capillary surface (the reaction should be almost instantaneous), before removing the Sigmacote liquid reagent with a gel loading pipette tip. Let remnants of the reagent evaporate overnight under a fume hood at RT. Flush the inside of the capillary with nuclease-free H_2_O instead of Sigmacote as described above, to wash out any potentially toxic remnants. Remove nuclease-free H_2_O and let all remaining fluid evaporate; pull medaka microinjection needles from the siliconized capillaries prior to injection.

2. Injection.

a. Inject the mix into 1-cell stage embryos as described previously [59] (Figures 3 and 4).

b. Injection parameters and needles must be calibrated to inject around 10%–15% of the cell volume for optimal efficiency and lethality; excess delivered volume will lead to drastic increases in lethality. For the more viscous Cas9 protein mix, it is likely necessary to increase the injection pressure relative to mRNA-based mix injections in order to easily reach the appropriate injected volumes mentioned above.

c. After injection, remove embryos from the agarose injection mold and transfer a maximum of 50 embryos per 10 cm diameter Petri dish containing 1× ERM. Incubate embryo dishes in an incubator set to 27 °C. Check for and remove dead embryos and change medium daily until embryos have hatched.

d. We found that for the KI of an eGFP donor into the *Cbx1b* gene (File S1), injections using Cas9 protein resulted in higher lethality than Cas9 mRNA, but also higher efficiency as judged by the number of embryos displaying visible fluorescent expression on average per 100 embryos injected (Table 1). However, both options are highly efficient for endogenous gene targeting.

**E. Screening and genotyping for data analysis**

*Notes:*

*1. Since Cas9 cutting and homology-directed repair might not occur at the 1-cell stage, but rather during later embryogenesis, F0s are likely mosaic, with some cells harboring KI alleles (Figures 3 and 4). Some cells might also harbor KO alleles, which can also be recovered if needed.*

*2. Because of the mosaic nature of genome editing in multicellular organisms, somatic editing does not necessarily correlate with germline transmission of the edit. Therefore, F0 genotyping can be performed to evaluate the editing efficiency, but PCR-genotyping of F1s derived from outcrosses of potential F0 founders is needed to confirm germline transmission.*

*3. F0s (potential founders) and F1s (several from each founder, since the germline transmission rate is variable) should be PCR-genotyped using primers annealing at ~250 bp from each side of the site of insertion site, in order to get a band of ~500 bp for the wildtype allele and ~1,200 bp for a standard-length fluorescent protein insertion allele. This ratio allows the amplification of the insertion allele even with the competition of the smaller wild-type allele. PCR bands corresponding to the insertion can be gel-purified and sequenced, or the PCR reaction can be directly purified and sequenced using primers annealing inside the fluorescent insert.*

*4. We recommend adding four controls to each fin clip experiment: a wild-type fish fin control as a positive control for the fin clip and gDNA extraction, a “no fin” negative control with just fin clip buffer that goes through the gDNA extraction process in parallel with the other samples, a positive control for the PCR reaction containing high-quality wild-type gDNA ideally extracted using a kit, and a no-template control containing nuclease-free H_2_O as a template instead of extracted gDNA. If a genotyping primer pair has not been tested before, we strongly recommend first running a gradient PCR on gDNA from a fin clip to determine optimal annealing temperatures. It is also advisable to check designed primer pairs for possible off-target amplifications using primerBLAST (* *https://www.ncbi.nlm.nih.gov/tools/primer-blast/* ; *check specificity to Japanese medaka (taxid:8090, Refseq representative genomes).*

1. Visual screening

a. Injected embryos are screened daily or at the expected stage of expression under a fluorescence stereoscope or by another imaging modality, not resulting in embryo death. Positive embryos are grown separately. Depending on the number of positive embryos, the brightest and most ubiquitously expressing ones are then grown to adulthood. In our experience, growing between 20 and 40 potential founders in total to adulthood is a good range for establishing a given line, but this varies a lot between KI designs.

b. If the fluorescent signal is too low to be detected in a visual manner, for instance, if it is restricted to a few cells, it might be difficult to identify positive fish with a visual screening. In this case, if other options such as using a brighter fluorophore donor are exhausted, it is possible to PCR genotype injected F0 fish grown to adulthood in order to identify fish with mosaic-edited alleles detectable by PCR. Germline transmission of correct insertion alleles in these founders has to then be confirmed by genotyping and sequencing of F1 embryos derived from outcrosses of F0 potential founders. Edited F1s can be raised to adulthood and fin-clip genotyped again to confirm the allele.

2. Fin-clipping and DNA extraction

a. Prepare tools and solution for fin-clipping: Prepare fresh fin-clip buffer by adding 50 μL of Proteinase K at 20 mg/mL to 1 mL of buffer (see Recipes). Dispense 50 μL of the prepared solution into labeled 1.5 mL tubes for each fish to be fin-clipped, and keep on ice. Prepare Tricaine anesthetizing solution by adding 5 mL of 0.4% Tricaine (see Recipes) to 100 mL of fish water. Prepare two containers, one containing 70% EtOH and one containing dH_2_O, for cleaning of scissors and forceps in between fin-clipping of individual fish. Clean the working area and the instruments used for fin-clipping with 70% EtOH before beginning (Figure 4).

b. Proceed as follows for fish fin-clipping:

i. Fully anesthetize individual fish by submerging them in Tricaine solution inside a net, until locomotion subsides and opercular movements are slowed but still present.

ii. Under a lamp, carefully cut a small piece of the caudal fin with scissors. Using forceps, transfer the piece to one of the tubes containing the fin-clip buffer. Be careful not to cut beyond halfway into the fin.

iii. Transfer individual fish into an adequate labeled container containing fish water and monitor proper recovery as judged by the return of spontaneous locomotion and regular opercular movements after a few minutes.

iv. Carefully clean and rinse forceps and scissors in between each fish with EtOH and dH_2_O.

v. Once all fish are fin-clipped, briefly spin down tubes and incubate at 65 °C for at least 4 h.

vi. Add 100 μL of nuclease-free H_2_O to each tube, mix, then transfer to 95 °C for 15 min to heat-inactivate the Proteinase K. Spin down tubes in a tabletop centrifuge at maximum speed for 1 min.

vii. Use 2 μL of the supernatant as a template for the genotyping PCR reaction.

3. PCR genotyping.

a. Assemble PCR genotyping reactions as follows (Table 9):

**Table 9.** Genotyping PCR mix

Component	Quantity
Fin-clip gDNA for each fish	2 μL
Forward genotyping PCR primer at 100 μM	0.25 μL
Reverse genotyping PCR primer at 100 μM	0.25 μL
2× Phusion or Q5 Master Mix	25 μL
Nuclease-free H_2_O	22.5 μL
A Master mix containing no template facilitates the PCR-genotyping of many fish	

b. Run reactions under the following standard genotyping conditions, adjusting the annealing temperature accordingly (Table 10):

**Table 10.** Genotyping PCR parameters

Step	Time and temperature
Initial denaturation	2 min at 98 °C
Denaturation step	30 s at 98 °C
Annealing step	30 s at the predicted or already determined optimal annealing temperature (from 60 to 72 °C)
Elongation step	45 s at 72 °C
Repeat denaturation/annealing/elongation steps for a total of 30 cycles	
Final elongation	10 min at 72 °C
Hold at 10 °C	

c. Run reactions on an agarose gel with a DNA dye and an appropriately sized DNA ladder until bands become visibly separable under UV transillumination (Figure 4).

d. PCR bands corresponding to the insertion can be gel-purified and submitted for Sanger sequencing. Alternatively, the PCR reaction can be purified directly and sequenced using primers annealing inside the fluorescent insert sequence facing outwards, as per the respective guidelines for Sanger sequencing sample submission and primer design.

4. Genome-engineered line establishment

a. Adult F0 possible founders are outcrossed individually to wildtype fish once they have reached sexual maturity (Figure 3), and F1 embryos are collected and screened daily as described above. We recommend screening at least 100 embryos per F0 fish, as we have observed germline transmission rates as low as 1% in individual F0, i.e., only 1 in 100 embryos produced by a F0 founder carrying the KI allele when screened visually.

b. Between 20–40 positive F1 embryos should be raised to adulthood, in order to secure the line and recover all, potentially differing, edited alleles containing perfect or imperfect insertions. F1 fish should be fin-clipped and sequenced individually to identify fish carrying a KI allele with desired sequence edits. These F1 fish are then used to establish a stable line through further outcrosses. After successful establishment of a given line, it can be informative to breed edited alleles to homozygosity, in order to observe for any gene loss-of-function phenotypes indicative of a hypomorphic allele.

c. We recommend not to disregard F1 fish with imperfect, i.e., not fully seamless, integration at this point, as in many cases, small genetic scars, such as homology arm duplications, can still lead to an in-frame functional fusion protein. This must, however, be tested through subsequent imaging and thorough functional characterization, both in the seamless integration and scar sequence cases (Figure 4, File S3).

## Validation of protocol

This protocol or parts of it has been used and validated in the following research article:

• Seleit et al. [6]. Endogenous protein tagging in medaka using a simplified CRISPR/Cas9 knockin approach, *eLife* (Figure 1, Figure 2, Supplementary file 3, and Supplementary file 4). Depending on the locus targeted and Cas9 used, the efficiency ranges from 7% to 59% (positive embryos/surviving injected embryos).

## General notes and troubleshooting

**Troubleshooting**

Problem 1: Linear donor PCR does not result in one clean band after purification.

Possible cause(s): Unsuitable primer sequences, PCR parameters.

Solution(s): Please refer to section D for detailed possibilities on troubleshooting primers and PCR conditions.

Problem 2: Cas9 protein-based microinjection mix frequently blocks the needle tips.

Possible cause(s): Protein precipitation, degradation in the assembled mix over time.

Solution(s): We recommend having a microcentrifuge nearby when injecting and briefly spinning down injection mix tubes prior to loading a new needle. It is advisable in general to prepare the mix soon before injection, for instance, in the time period after putting male and female fish together again, and not using mixes kept at RT for longer than 2 h.

Problem 3: Microinjection of Cas9 protein or mRNA mix leads to very high embryo lethality.

Possible cause(s): Volume delivered through microinjection is too high, concentration of components inside the mix is too high, the targeted gene has a lethal phenotype.

Solution(s): Injection parameters (i.e., injecting pressure, holding pressure, injection time) need to be calibrated to deliver the right volume of around 10%–15% of the zygote. In our experience, especially the more viscous protein mix will need different injection parameters from routine aqueous solution conditions in order to carry out reasonable injections.

Concentrations of all components should be measured and precise to the concentrations described in this protocol, and of high purity. This is particularly important for any gel-extracted PCR donors, which are known to have high contaminant carryover. It is possible to inject the components of a mix individually, in order to rule out one component causing high toxicity.

Should a protein-based mix lead to very high lethality, it can be worthwhile to inject Cas9 protein and guide RNA without the repair donor, in order to assess the lethality of likely KO alleles for a particular experiment design. If high lethality persists, it is worthwhile to switch to an mRNA-based mix in order to try to recover an allele with a correct integration. In some KI donor cases, it can also be worthwhile testing a linear DNA donor concentration of 20 ng/μL instead of 40 ng/μL when using Cas9 protein, and 5 ng/μL instead of 10 ng/μL when using Cas9 mRNA, since this will reduce the toxicity associated with linear dsDNA donors. It is also possible to reduce the quantity of Cas9 protein or mRNA if KO and/or KI alleles have deleterious effects.

Problem 4: KI mix leads to no or very low numbers of positive fish.

Possible cause(s): mRNA/protein or guide RNA are degraded or inefficient, fluorescent protein-tagged gene expression is too low for stereoscope-based visual screening, incorrect injection technique, or a particular experiment design yields low efficiency “by design.”

Solution(s): In order to assess the efficiency of mRNA or protein batches, test KO injections can be performed against an easily phenotypable gene such as *oca2* for loss of pigmentation in embryos [28]. It is advisable to do this once right after the synthesis of a new batch of mRNA or the arrival of a commercial shipment of Cas9 protein, in order to establish a baseline to compare batch quality over storage time.

Should a gene be expected to be lowly expressed or expressed in a small number of cells under specific timing or stimuli, such as many transcription factors, it is possible to raise embryos without visual screening and fin-clip adult F0 fish. In this case, we strongly recommend, however, to co-inject with a fluorescent marker as described previously [59] or a visible dye such as 0.02% phenol red [61] in order to identify correctly injected embryos. This is especially important for less experienced injectors and can also be used to assess the number of correctly injected embryos. An experienced injector would be able to get more than 90% of embryos with fluorescence when injecting with a fluorescent injection marker, and the numbers presented in this protocol rely on a comparable injection efficiency.

If it is not possible to retrieve any positives either through visual screening or genotyping for a given experiment design after having gone through extensive troubleshooting, then the KI design might need to be changed. It is possible that a particular protein fusion alters the endogenous protein function, leading to non-viable embryos, even in a mosaic/heterozygous condition. In this case, we recommend using another fluorescent reporter or performing the KI in another position in the gene sequence. We also recommend to aim for an insertion as close as possible to the Cas9 cut, and therefore to choose a guide RNA accordingly, even if this guide RNA might have a low GC content or might generate off-target cutting in non-coding sequences (in this case, the off-target sites may need to be sequenced after the generation of the KI line). It is also possible that a KI design is inefficient due to single-nucleotide polymorphisms (SNPs) at the targeted locus, and it is advisable to sequence the locus to determine if the design needs to be adapted.

## Supplementary information

The following supporting information can be downloaded here:

1. File S1. Design example (cbx1b with eGFP KI).

2. File S2. Fluorescent reporter plasmid sequences.

3. File S3. Confocal test imaging of a *marpe1b(eb1)-mScarlet* Knock-In reporter line [6], labeling microtubule plus-ends. Shown is a side-view timelapse of a stage-10 early blastula medaka embryo, with synchronous divisions of blastomeres and the yolk syncytial layer [18] visible.
